# Thermodiffusion of Chained Molecules: From Oligomers to the High Polymer Limit

**DOI:** 10.1021/acs.langmuir.5c01186

**Published:** 2025-05-30

**Authors:** Konstantin I. Morozov, Werner Köhler

**Affiliations:** † Department of Chemical Engineering, 26747 Technion—Israel Institute of Technology, Haifa 32000, Israel; ‡ Physikalisches Institut, 26523 Universität Bayreuth, D-95440 Bayreuth, Germany

## Abstract

Thermodiffusion of entangled molecules in an inhomogeneous temperature field is determined by their length. With increasing length, the thermophoretic velocity increases in magnitude and sometimes changes its direction, depending on the nature of the polymer and the solvent. Thus, the theoretical description of thermodiffusion, already a multifactorial phenomenon, is complicated by the appearance of another important property. Here, we generalize to chained molecules an approach recently proposed for molecular systems and calculate the thermodiffusion coefficients of polymer molecules, starting from oligomers up to the high polymer limit. The calculations were performed for two types of chained molecules—polystyrene (PS) and alkanes—dissolved in one of three nonpolar solvents—toluene, ethylbenzene, or cyclohexane. For both types of chain molecules, the thermodiffusion coefficient *D*_*T*_ saturates with length, but with different scaling exponents. The predicted values of *D*_*T*_ of PS in toluene and ethylbenzene are in excellent agreement with experimental data over the entire range of chain lengths from monomer to high polymer. In particular, the plateau value of the product η*D*_*T*_ (where η is a solvent viscosity) proves to be close to the experimentally observed universal value ≈ 6 · 10^–15^ N/K. The theoretical dependencies for *D*_*T*_ of alkanes in the same solvents also agree well with the data, although they slightly overestimate it. More significant deviations of the predictions from the measurements occur when cyclohexane is used as a solvent. This behavior is similar to that found earlier for molecular mixtures. In all solvents studied, alkane molecules manifest the negative values of the thermodiffusion coefficients and migrate to the hotter layers.

## Graphical abstract

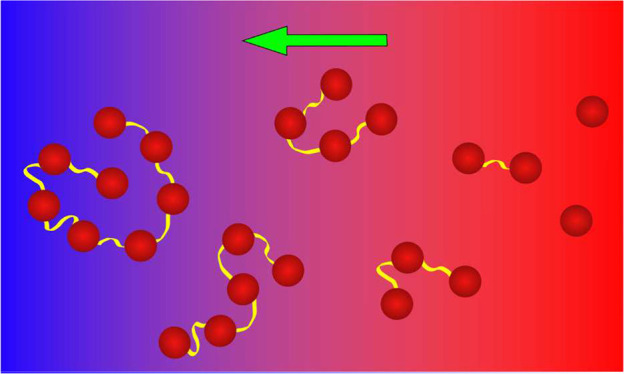

## Introduction

The thermophoretic behavior of the separation of substances in a multicomponent mixture initiated by temperature gradients is known as thermodiffusion or Soret effect.^1,2^ The phenomenon is observed in many different systems such as gaseous and liquid mixtures, colloids and polymer solutions.^3,4^ In the dilute limit, the thermodiffusion drift velocity of the minority component is given by ***v***_*T*_ = – *D*_*T*_∇*T*, where *D*_*T*_ is the thermodiffusion coefficient.^4^ One of the most striking properties of thermodiffusion in polymer solutions is the independence of ***v***_*T*_ and *D*_*T*_ from the polymer chain length in the case of *sufficiently long* polymer molecules. Since its discovery in a solution of polystyrene (PS) in toluene,^5^ the effect has been repeatedly observed in solutions of various polymers and solvents^6–13^ and is now well documented.

The thermophoretic behavior of *shorter* chains turns out to be completely different from that of long chains.^11,14–16^ Rauch, Stadelmaier and Köhler in their systematic studies^11,14^ of the molar mass dependence of *D*_*T*_ of PS in different solvents showed that *D*_*T*_ becomes chain length dependent for oligomers and shorter polymer chains. In the high polymer limit, the quantity η*D*_*T*_, where η is the solvent viscosity, proves to be constant for all solvents and all degrees of polymerization. However, for shorter chains *D*_*T*_ decreases with molar mass and for some solvents, e.g., cyclohexane or cyclooctane, it even changes its sign.^14^ The transition of *D*_*T*_(*M*) from the short chains to the molar mass independent plateau occurs when the size of the polymer is about the Kuhn segment.^14,15^ The same conclusion was reached a little earlier by Zhang and Müller-Plathe in a nonequilibrium molecular dynamics modeling of polymer thermodiffusion.^17^ In refs 15, 18 , it was also shown that for the stiffer polymers the product η*D*_*T*_(*M* → *∞*) is not only solvent-independent but also independent of the *type of polymer* in the high polymer limit, taking the universal value η*D*_*T*_^*∞*^≈ 6 · 10^–15^ N/K.

A first theoretical explanation of the molar mass independence of *D*_*T*_ was given by Brochard and de Gennes,^19^ who assumed the absence of long-range interactions between monomers and applied the Rouse model, describing the polymer as a draining coil with independent motion of the segments. Since ref 19, the Rouse model has been taken for granted in almost all theoretical investigations^20–23^ of thermodiffusion in polymer solutions. It is interesting that in the description of the usual (Fickian) diffusion, the polymer coil was considered as a nondraining one, in agreement with the Zimm model. Thus, for more than 30 years, the permeability of the polymer coil through the solvent was considered as a kind of centaur: the coil was nondraining with respect to diffusion motion and draining with respect to thermodiffusion.

By the early 2010s, strong experimental evidence against Rouse’s picture for polymer thermodiffusion had emerged. In refs 24–26 , the thermoresponsive polymer poly­(*N*-isopropylacrylamide) (PNIPAM) was studied, which undergoes a coil–globule transition at a temperature of 32 °C, with the expanded coil at lower and the compact globule at higher temperatures. The polymer was used in three modifications—as linear polymer^24^ and as cross-linked microgel spherical particles^25^ or shells grafted the polystyrene colloidal particles.^26^ It was shown that below the coil–globule transition the behavior of *D*_*T*_ of the linear polymer and the core–shell particle is almost identical.^26^ Moreover, the *D*_*T*_ of the microgel proved to be completely insensitive to the chain collapse into a globular state.^25^ Finally, let us also mention the very first observation questioning the Rouse picture. In ref 9, Schimpf and Giddings, studying thermodiffusion of block copolymers, showed that *D*_*T*_ is determined by the monomers located in the outer region of the coil.

Based on these data, it was concluded^27^ that similar to Fickian diffusion, thermal diffusion of polymers also takes place according to the Zimm picture of a nondraining coil, where the solvent is immobilized within the inner regions of the coil. As a result, only the monomers from the thin outer (draining) layer participate in the thermodiffusion motion. By analyzing the tangential stresses within this layer,^27^ it has been shown that the thermophoretic velocity of the polymer coil is of the same order of magnitude as that of the Kuhn segments. This implies, first, the molar mass independence of *D*_*T*_ within the framework of the Zimm mechanism. Second, the Kuhn segment proves to be an approximate molecular size at which the crossover from small molecule to polymeric behavior in the Soret effect occurs. The latter is in full agreement with the experimental data.^14–16^

The theoretical determination of the thermodiffusion coefficient *D*_*T*_ and the Soret coefficient *S*_*T*_ = *D*_*T*_/*D* (where *D* is the diffusion coefficient) is a challenging problem. Recently, one of us proposed a new approach to calculate the Soret coefficient *S*_*T*_ of simple binary mixtures.^28^ The Soret coefficient was written as the sum of the chemical and kinetic contributions. The former is related to interparticle interactions and is attributed to the gradient of a partial pressure of one of the mixture components.^29^ As equilibrium thermodynamic properties, the partial pressures and thus the chemical contribution to the Soret coefficient are successfully described within the framework of the perturbed-chain statistical associating fluid theory (PC-SAFT).^30^ The kinetic contribution due to intermolecular scattering phenomena is a nonequilibrium property. It is approximated by a relation associated with hydrodynamic fluctuations.^31^ The predicted values of the Soret coefficient of equimolar binary mixtures have been compared with the experimental data available for 113 mixtures composed of 25 nonassociating and non or weakly polar simple liquids. It was found a good agreement of both data, especially in the particular case of the alkane family.^28^

The present study deals with the dilute binary mixtures of *chained molecules* of polymers or alkanes in molecular solvents. Chained component 1 is assumed to be of low concentration and is hereafter referred to as the solute. Here we apply the formalism proposed in ref 28, for the calculation of the thermodiffusion coefficients of such systems. Our goal is to find out how the thermodiffusion coefficients change with increasing polymer chain length, starting from oligomers. We would like to clarify to what extent the model allows to describe the universal behavior of *D*_*T*_ of polymer systems and the special behavior of the alkane family. The paper is organized as follows. First, we present the basic steps for calculating the chemical and kinetic contributions according to ref 28. Then we determine the model parameters of the alkane/polymer series and calculate the thermodiffusion coefficients of their solutions in three nonpolar solvents. For PS solutions in toluene and ethylbenzene, we find an excellent agreement of the predictions with the experimental data over the whole range of the chain length. Finally, we summarize our results and draw conclusions.

## Theory

### Calculation of the Soret Coefficient and Selection of the Polymer Systems

In this section we will review the main steps in calculating the Soret coefficient for dilute binary mixtures given in.^28^ The Soret coefficient is represented as the sum of the chemical and kinetic contributions:

$$S_{T}=S_{T}^{\left(\mathrm{chem}\right)}+S_{T}^{\left(\mathrm{kin}\right)}$$ 1

The chemical contribution *S*_*T*_^(chem)^ is due to the intermolecular interactions. According to refs 29, 32 , it is related to the temperature gradient of the partial pressure of the solute in the solvent and can be written in the form:^28^

$$S_{T}^{\left(\mathrm{chem}\right)}=\frac{1}{n_{2}T}{\left[\frac{\partial (n_{2}TZ_{12})}{\partial T}\right]}_{p}$$ 2

where *n*_2_ is the number density of the solvent molecules, *T* is the temperature, *p* is the normal pressure, and *Z*_12_ is the compressibility factor of the solute in the solvent, defined over the solute partial pressure *p*_1_ as

$$p_{1}=n_{1}k_{B}TZ_{12}$$ 3

with the Boltzmann constant *k*_*B*_.

The solute compressibility *Z*_12_ is determined within the framework of the PC-SAFT model. In the case of nonassociating and nonpolar pure liquids, the PC-SAFT model deals with three temperature independent parameters: the segment number *m*, the hard core segment diameter σ and the segment–segment interaction parameter ϵ.^30^ The parameters are found by fitting to some experimental data. As a rule, their values obtained in different sources^30,33–35^ agree well with each other. By the expression ([Disp-formula eq2]) the model parameters *m*, σ, and ϵ determine the chemical contribution *S*_*T*_^(chem)^ to the Soret coefficient of the solute.

The kinetic contribution *S*_*T*_^(kin)^ is due to collisions and scattering processes between the molecules. It is represented as the sum of the gas term and the liquid fluctuation contribution^28^

$$S_{T}^{\left(\mathrm{kin}\right)}=S_{T}^{\left(\mathrm{gas}\right)}+S_{T}^{\left(\mathrm{fluc}\right)}$$ 4

The gas term is taken in the Chapman–Enskog form:^36,37^

$$S_{T}^{\left(\mathrm{gas}\right)}=\frac{105}{118}\frac{M_{1}-M_{2}}{M_{1}+M_{2}}\frac{1}{T}$$ 5

where *M*_1_ and *M*_2_ are the molar masses of the components.

The liquid fluctuation term is written in analogy to the Brownian thermal drift term^31^ and is proportional to the temperature derivative of the solvent viscosity η:

$$S_{T}^{\left(\mathrm{fluc}\right)}=f(\rho_{1}/\rho_{2})|d(\ln\;\eta )/dT|$$ 6

where the function *f*(ρ_1_/ρ_2_) depends only on the ratio of the component densities. The explicit form of the function is^28^

$$f(\rho_{1}/\rho_{2})=C_{1}\sqrt{1-\rho_{2}/\rho_{1}}+C_{2}(1-\rho_{2}/\rho_{1}),\;\rho_{1}>\rho_{2}$$ 7

$$f(\rho_{1}/\rho_{2})=-C_{1}\sqrt{\rho_{2}/\rho_{1}-1}+C_{2}(1-\rho_{2}/\rho_{1}),\;\rho_{1}<\rho_{2}$$ 8

The constants *C*_1_ and *C*_2_ were obtained by fitting ([Disp-formula eq6]) to the Soret coefficient data in mixtures with a predominant kinetic contribution:

$$C_{1}=0.2327,\;C_{2}=0.4193$$ 9

The relative weight of both contributions—chemical and kinetic—was compared for 141 mixtures.^28^ They were found to be of the same order of magnitude and thus of fundamental importance in the calculation of the Soret coefficient. Comparison of the predicted values of the Soret coefficient with experimental data showed good agreement, especially in the case of nonpolar components. However, taking into account the dipole moments of the molecules within the framework of the polar PC-SAFT model^38^ did not lead to a noticeable improvement of the results. In our opinion, this is due to the fact that the existing PC-SAFT models^30,33–35,38^ do not take into account the permittivity of liquids when fitting parameters to experimental data. [The difficulties of considering the permittivity of fluids in numerical modeling are recently discussed in ref 39.] This makes the thermodynamic description of polar liquids not completely self-consistent, leading to a noticeable distortion of the dipole contributions to the Soret effect.

Therefore, in the following we will limit ourselves to considering only the nonpolar systems—polymers with nonpolar elementary units dissolved in nonpolar molecular liquids. As the latter, we will take three liquids: toluene, cyclohexane or ethylbenzene. Note that thermodiffusion with the first two liquids has already been studied in ref 28 in the case of molecular mixtures. Polymers suitable for our needs are polystyrene (PS) and polyethylene (PE). As mentioned above, there is an extensive bibliography on thermodiffusion in solutions of PS and *n*-alkanes (PE oligomers), see refs 5–9, 11, 12, 14–16, 18 . According to refs 14, 15 , both polymers have quite different chain flexibilities with Kuhn segment masses differing by more than a factor of 4: about 1000 for the stiffer PS as compared to 230 for the very flexible PE. Thus, the Kuhn segment of alkanes is comparable in size to a solvent molecule, while the Kuhn segment of PS is much larger. As we will see below, this ultimately leads to thermodiffusion drift in opposite directions in both systems.

### Model Parameters for Alkane/Polymer Series and Solvents

To calculate the Soret coefficient using the formulas in the previous section, we need to know the parameters of the PC-SAFT model: *m*, σ, and ϵ. For *n*-alkanes the parameters are well-known.^30,33–35^ In the case of PS, the problem is less defined since there is no data on the equation of state for PS oligomer systems. The only exception is *ethylbenzene*, which corresponds to the effective repeat unit of PS.

Here we want to describe both series of polymers in a unified way. To do this, we will use the strategy proposed by Kontogeorgis et al. in refs 40, 41 . First, we write the functional form between the parameters *m*, σ and ϵ and the molecular weight MW of the polymer found for the alkane series:

$$m=A_{m}\mathrm{MW}+B_{m}$$ 10

$$m\sigma^{3}=A_{\sigma }\mathrm{MW}+B_{\sigma }$$ 11

$$m\epsilon /k_{B}=A_{\epsilon }\mathrm{MW}+B_{\epsilon }$$ 12

Fitting these relations to the PC-SAFT parameters reported by Gross et al. in ref 33 for linear alkanes from ethane (C_2_H_6_) to octacosane (C_28_H_58_) yields the unknown coefficients *A* and *B* given in Table 1.

**Table 1:** Summary of the Coefficients in Eqs ([Disp-formula eq10])–([Disp-formula eq12]) and Average Absolute Deviations between Calculated and Experimental Densities for Alkane and PS Series

substance	*A*_*m*_ [−]	*A*_σ_ [Å^3^]	*A*_ϵ_ [K]	*B*_*m*_ [−]	*B*_σ_ [Å^3^]	*B*_ϵ_ [K]	AAD [%]
alkanes	0.02672	1.708	7.133	0.8109	19.98	106.4	0.5
PS	0.02050	1.467	7.138	0.9141	11.30	129.5	0.6

Figure 1 shows the linear dependence of the parameter combinations *m*, *m*σ^3^, and *m*ϵ/*k*_*B*_ on the molecular weight of alkanes.

**Figure 1:**
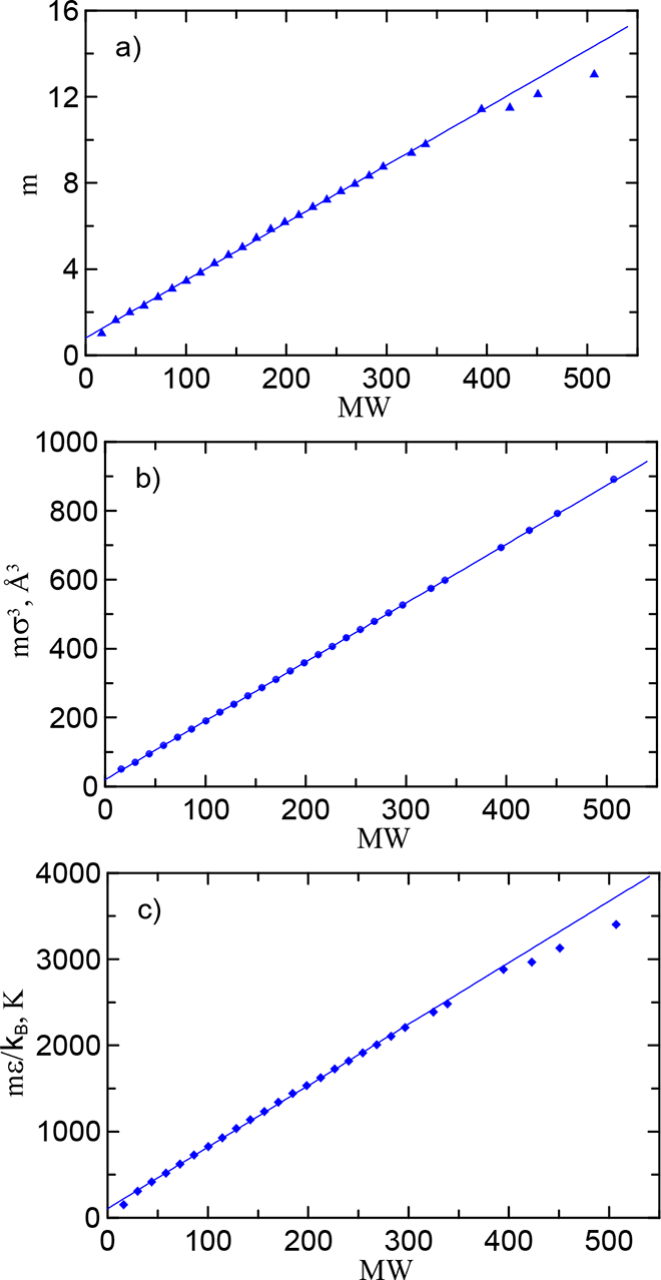
Model parameters *m*, *m*σ^3^, and *m*ϵ/*k*_*B*_ vs molecular weight for linear alkanes. Symbols are the PC-SAFT values reported in ref 33. The linear fit ([Disp-formula eq10])–([Disp-formula eq12]) from ethane (C_2_H_6_) up to octacosane (C_28_H_58_) corresponds to the alkane coefficients from Table 1.

Furthermore, after^40,41^ we assume that the eqs ([Disp-formula eq10])–([Disp-formula eq12]) hold for *all* polymers. The coefficients *A*_*m*_, *A*_ϵ_, and *A*_σ_ are determined by fitting to experimental data in the high polymer limit (MW → *∞*). In contrast to molecular systems, they are more scattering. Here we use the values obtained in ref 41 : *m*/MW = 0.0205, σ^*∞*^ = 4.152 Å, ϵ^*∞*^/*k*_*B*_ = 348.2 K which leads to

$$A_{m}^{\left(\mathrm{PS}\right)}=0.0205,\;A_{\sigma }^{\left(\mathrm{PS}\right)}=1.467\;{Å}^{3},\;A_{\epsilon }^{\left(\mathrm{PS}\right)}=7.138\;K$$ 13

The constants *B*_*m*_^(PS)^, *B*_σ_^(PS)^, and *B*_ϵ_^(PS)^, which describe the influence of the end groups for oligomers and short polymer chains, are found by fitting the eqs ([Disp-formula eq10])–([Disp-formula eq12]) to the model parameters for ethylbenzene (ebz): MW_ebz_ = 106.18, *m*_ebz_ = 3.0887, σ_ebz_ = 3.7810 Å, ϵ_ebz_/*k*_*B*_ = 287.08 K.^35^ Finally we get

$$B_{m}^{\left(\mathrm{PS}\right)}=0.9141,\;B_{\sigma }^{\left(\mathrm{PS}\right)}=11.30\;{Å}^{3},\;B_{\epsilon }^{\left(\mathrm{PS}\right)}=129.5\;K$$ 14

All coefficients for alkane and PS series are collected in Table 1. The last column shows the average percentage absolute deviations between the PC-SAFT model-calculated and experimental liquid density values.

Finally, we give the model parameters for three molecular solvents—see in Table 2.

**Table 2:** Summary of Pure Solvent Parameters

substance	*m* [−]	σ [Å]	ϵ/*k*_*B*_ [K]	ref	–d(ln η)/d*T* · 10^2^ [K^–1^]	ref
toluene	2.7579	3.7366	289.36	^33^	1.20	^42^
ethylbenzene	3.0887	3.7810	287.08	^35^	1.24	^43^
cyclohexane	2.4870	3.8574	281.19	^33^	1.66	^44^

In general, all parameters of the three solvents are quite close in magnitude. Therefore, it is to be expected that the calculated values of the thermodiffusion coefficients *S*_*T*_ and *D*_*T*_ in all three liquids will also be close to each other. However, the results for the first two solvents, toluene and ethylbenzene, which differ in only one methylene group, are expected to be particularly close.

At the end of this section we would like to comment on the special case of cyclohexane as a solvent. In ref 28, when studying mixtures of 25 molecular liquids, it was found that the theoretical predictions of the model differ maximally from the experimental data when the solvent is represented by sufficiently large cyclic molecules. A possible reason for this is the overestimation of the kinetic contribution ([Disp-formula eq4]). Therefore, among the three solvents, the most significant deviations of the predictions from the experimental data are expected when cyclohexane is used as solvent.

## Results

### Alkane Series

First, we consider the thermodiffusion coefficients of alkanes dissolved at infinite dilution in three solvents—toluene, ethylbenzene, or cyclohexane. The experimental values of *S*_*T*_ of the mixtures are taken from refs 15, 45–47 . They are shown by symbols in Figure 2 for the alkane series. As can be seen, the data found for different solvents prove to be close to each other and increase by (1.5 – 2) · 10^–3^ K^–1^ with the growth of the alkane chain from hexane to hexadecane.

**Figure 2:**
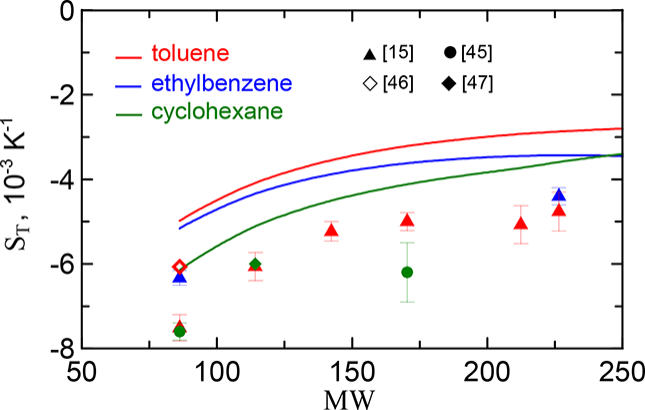
Soret coefficients of alkanes at infinite dilution in three solvents vs molecular weight. The measured (symbols) and theoretical (solid lines) values for toluene, ethylbenzene and cyclohexane are shown, correspondingly, by red, blue, and green. The inset shows the references of the data marked with symbols. The theoretical curves are plotted according to eqs ([Disp-formula eq1]), ([Disp-formula eq2]), ([Disp-formula eq5])–([Disp-formula eq9]). The model parameters for solute and solvents are taken from Tables 1 and 2.

The theoretical dependencies of the Soret coefficient are determined according to eqs ([Disp-formula eq1]), ([Disp-formula eq2]), ([Disp-formula eq5])–([Disp-formula eq9]) with the solute and solvent model parameters from Tables 1 and 2. The predicted values overestimate the experimental data by about 2 · 10^–3^ K^–1^. At the same time, similar to the experiment, they turn out to be close for three solvents and grow almost parallel to the data with alkane weight.

Let us consider the individual contributions to the Soret coefficient *S*_*T*_. Following the general methodology of PC-SAFT,^30^ we split the chemical contribution *S*_*T*_^(chem)^ into the hard chain reference contribution *S*_*T*_^(hc)^ and the dispersion interaction contribution *S*_*T*_^(disp)^. Then the eq 1 for the Soret coefficients takes the form

$$S_{T}=S_{T}^{\left(\mathrm{hc}\right)}+S_{T}^{\left(\mathrm{disp}\right)}+S_{T}^{\left(\mathrm{kin}\right)}$$ 15

Let us consider these three contributions in the case of a particular mixture of alkane series in toluene. The Soret coefficient of this family is represented by the red curve in Figure 2. In Figure 3 it corresponds to the black curve, while the individual components are given by colored lines. The hard chain contribution *S*_*T*_^(hc)^ shown by the blue line was calculated using eq 2 for the chemical contribution, but with the dispersion solute–solvent interactions switched off, ϵ_12_ = 0. The dispersion interaction contribution *S*_*T*_^(disp)^ shown by the red is the difference between the chemical and hard chain contributions, *S*_*T*_^(disp)^ = *S*_*T*_^(chem)^ – *S*_*T*_^(hc)^. The kinetic contribution (see green curve in Figure 3) was determined according to eqs ([Disp-formula eq4])–([Disp-formula eq9]).

**Figure 3:**
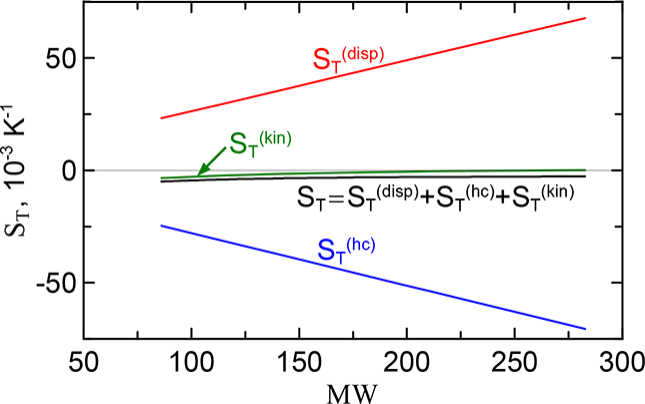
Full Soret coefficient and its individual contributions for alkanes at infinite dilution in toluene vs solute molecular weight. The red, blue, and green lines mark the dispersion, hard-chain and kinetic contributions, respectively. The black solid line is the full Soret coefficient.

Several characteristic features of the behavior of the contributions to the Soret coefficient can be noted. (i) The dispersion and hard chain contributions have different signs and largely offset each other. However, the negative hard chain contribution dominates, since its sum is negative. Its absolute value is about one order of magnitude smaller than each of the contributions. (ii) The kinetic contribution increases with the molecular weight of the alkane, which finally determines a slight increase of the total coefficient *S*_*T*_ with MW. From this it can be concluded that in the case of alkanes the chaining of segments leads to an increase of the Soret coefficient. As we will see below, the similar property also holds for PS. (iii) On the scale of the figure all contributions look like almost perfect straight lines. This is not the case, however, if we look at the total value of *S*_*T*_ in Figure 2, which is drawn on a more detailed scale.

To calculate the thermodiffusion coefficient *D*_*T*_ = *S*_*T*_ *D*, we need to know the values of the limiting diffusion coefficient *D* of *n*-alkanes in three solvents. In Figure 4 by red symbols there are shown experimental data^15^ on limiting diffusion coefficients of six alkanes—hexane, octane, decane, dodecane, pentadecane, hexadecane, and eicosane—in toluene as a function of alkane molecular weight. The data are well described by the dependence

$$D=D_{\mathrm{hex}}({\mathrm{MW}}_{\mathrm{hex}}/\mathrm{MW}{)}^{1/2}$$ 16

where MW_hex_ and *D*_hex_ are the molar mass and diffusion coefficient of hexane. In the following we use the relation ([Disp-formula eq16]) to calculate the diffusion coefficients *D* of alkane series. The values of the diffusion coefficient *D*_hex_ of hexane in three solvents—toluene, ethylbenzene, and cyclohexane—are *D*_hex/tol_ = 24.9 · 10^–10^ m^2^s^–1^,^15^ *D*_hex/ebz_ = 22.0 · 10^–10^ m^2^s^–1^,^15^ and *D*_hex/chx_ = 16.8 · 10^–10^ m^2^s^–1^.^45,48^ The corresponding curves for limiting diffusion in alkane/ethylbenzene and alkane/cyclohexane mixtures are shown in Figure 4 with the data from ref 12, 15, 49, 50 . Note that the value of *D* for heptane-ethylbenzene obtained in^49^ at *T* = 313.15 K was recalculated to *T* = 298.15 according to the rule: *D* ∼ *T*/η_ebz_, where η_ebz_ is the viscosity of ethylbenzene.

**Figure 4:**
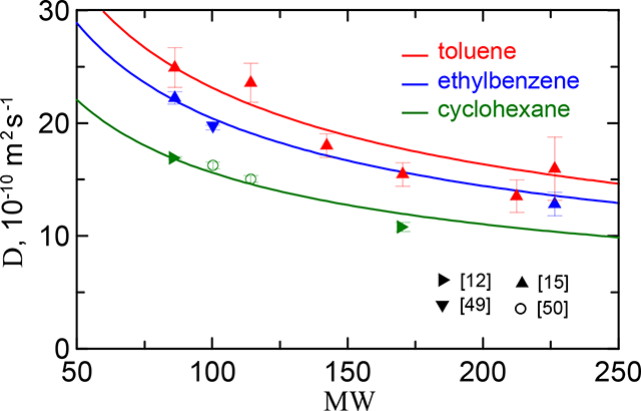
Diffusion coefficient of *n*-alkanes at infinite dilution in toluene as a function of molecular weight. The symbols mark the data from.^12,15,49,50^ The theoretical curves satisfy eq 16 with *D*_hex/tol_ = 24.9 · 10^–10^ m^2^s^–1^, *D*_hex/ebz_ = 22.0 · 10^–10^ m^2^s^–1^, and *D*_hex/chx_ = 16.8 · 10^–10^ m^2^s^–1^.

In the high polymer limit, the theoretical predictions for *S*_*T*_ and the formal substitution of eq 16 into *D*_*T*_ lead to a dependence with a maximum at MW ∼ 400—see the dashed curve in Figure 5. When constructing the curve, we did not take into account the property discussed in the introduction: the dependence *D*_*T*_(MW) saturates when the chain size is about the Kuhn segment. As mentioned above, the value of the Kuhn segment mass of PE is about 230,^15^ corresponding to hexadecane (hxd) in the alkane series. An appropriate parametrization for *D*_*T*_ is^15^

$$D_{T}=\Delta_{T}/\eta -a/{\mathrm{MW}}^{\alpha }$$ 17

where Δ_*T*_, *a*, and α are constants and η is the solvent viscosity. We determine three constants that fit Eq. 17 to the theoretical values of *D*_*T*_ = *S*_*T*_ *D* found for alkanes from hexane to hexadecane. The exponent α = 2 proves to be an excellent parametrization in the given molecular weight range. The values of the parameters Δ_*T*_ and *a* found for three solvents are given in Table 3.

**Table 3:** Solvent Parameters Δ_*T*_ and *a* and Exponent α as Obtained Fitting Eq 17 to Predicted Values of *D*_*T*_ for Alkanes at MW_hex_ ≤ MW ≤ MW_hxd_ and for PS at MW_ebz_ ≤ MW ≤ 1000

polymer	solvent	Δ_*T*_/[10^–15^ N/K]	*a*/[10^–10^ m^2^/s]	α
PE	toluene	–1.873	692.5	2
	ethylbenzene	–2.415	590.3	2
	cyclohexane	–2.772	571.7	2
PS	toluene	6.733	12.69	1
	ethylbenzene	6.524	10.66	1
	cyclohexane	8.209	8.281	1

**Figure 5:**
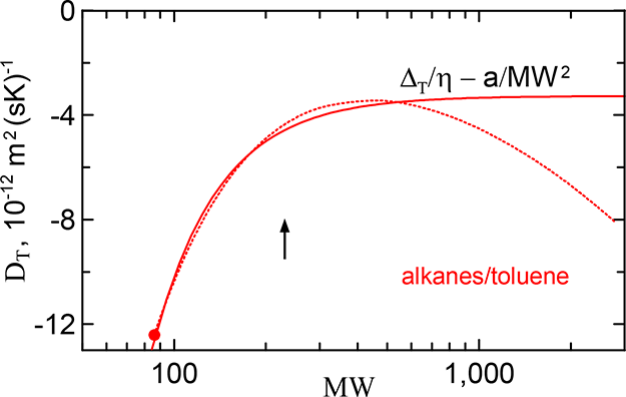
Thermodiffusion coefficient *D*_*T*_ of *n*-alkanes at infinite dilution in toluene as a function of molecular weight. The solid line is the parametrization ([Disp-formula eq17]). The arrow marks the molecular weight of the Kuhn segment. The theoretical dotted curve is depicted according to eqs ([Disp-formula eq1]) and ([Disp-formula eq16]) without taking into account the saturation of *D*_*T*_ at MW ≈ MW_hxd_. The circle marks the molecular weight of hexane.

A remarkable property of the parameters is that they are close to each other for all solvents. As a consequence, the three dependencies η*D*_*T*_—see Figure 6—are also close to each other, approaching their common asymptotic value in the high polymer limit: η*D*_*T*_^*∞*^ ≈ −(2 – 2.5) · 10^–15^ N/K.

**Figure 6:**
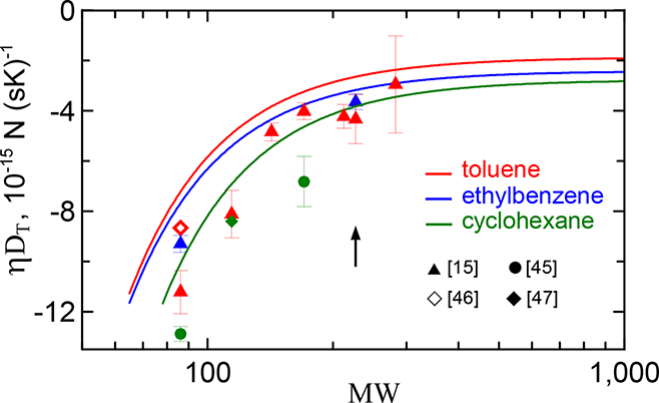
Product η*D*_*T*_ for *n*-alkanes in three solvents. The solid lines are the parametrization ([Disp-formula eq17]) with α = 2 and parameters from Table 3. The arrow marks the molecular weight of the Kuhn segment. The symbols are the experimental data as in Figure 2. Additionally it is included the data^15^ for eicosane shown by the most right symbol with maximal error bar.

The theoretical dependencies shown in Figure 6 explicitly demonstrate the transition in the behavior of *D*_*T*_ from PE oligomers and short alkane chains to long molecules. All dependencies grow almost parallel to each other with molecular weight and saturate to close values beyond the alkane Kuhn mass MW_Kuhn_ = 230. The theoretical dependencies are in good agreement with the experimental data, although they slightly overestimate them, as can be seen from Figure 6. The maximum deviation occurs in the case of cyclohexane, in accordance with our expectations made above.

To conclude this section, we note why the values of *D*_*T*_ turn out to be negative for all values of alkane molecule length. According to ref 28, the Soret coefficients are particularly sensitive to the values of the model parameters σ and ϵ. Let us estimate the values of these parameters for hexadecane, which determines the size of the Kuhn segment of alkanes. As mentioned above, starting from this size, the thermophoretic mobility *D*_*T*_ practically does not change. According to eqs ([Disp-formula eq11]), ([Disp-formula eq12]) and Table 1, σ_hxd_ = 3.90 Å and ϵ_hxd_/*k*_*B*_ = 251 K. The former value is quite close to that of the solvents—see Table 2. The latter, however, turns out to be 30–40 K lower than the ϵ values for the solvents. As a result, hexadecane as a liquid with lower values of the interaction parameter ϵ migrates to warmer layers of the mixture. The same conclusion can be drawn for shorter alkanes, since *D*_*T*_ decreases with shortening of the alkane molecules. Therefore, thermodiffusion of alkanes of any length is characterized by negative values of the coefficients *S*_*T*_ and *D*_*T*_.

### PS Series

As mentioned above, the plateau of *D*_*T*_ is the most brilliant property of thermodiffusion of polymer solutions in the high polymer limit. To calculate *D*_*T*_ = *S*_*T*_ *D* within our approach, one needs to know the values of the translational diffusion coefficient *D* of PS oligomers with molecular weight MW ≤ MW_Kuhn_^PS^. The latter is the mass of the PS Kuhn segment about 1000.^15^ For toluene, the diffusion coefficient of the oligomers is correctly described by the relation^12^

$$D=\Gamma /\sqrt{\mathrm{MW}}\cdot 10^{-9}\;m/s^{2}$$ 18

The fitting value of the coefficient is Γ_tol_ = 16.8.^12^ In the following we use the relation ([Disp-formula eq18]) also for the cases of ethylbenzene and cyclohexane as solvents. To the best of our knowledge, there are very few experimental data on translational diffusion of oligomers in these solvents. We have determined the unknown coefficients Γ for these solvents based mainly on the data found in ref 15 for the oligomer with mass MW = 370 and characterized by the smallest scatter. The fitting values are Γ_ebz_ = 17.3 and Γ_chx_ = 11.5. Note that the ratios Γ_ebz_/Γ_tol_ = 1.03 and Γ_chx_/Γ_tol_ = 0.68 are somewhat different from the corresponding inverse viscosity ratios, η_tol_/η_ebz_ = 1.15 and η_tol_/η_chx_ = 0.61. The comparison of the dependencies ([Disp-formula eq18]) with the experiment is shown in Figure 7.

**Figure 7:**
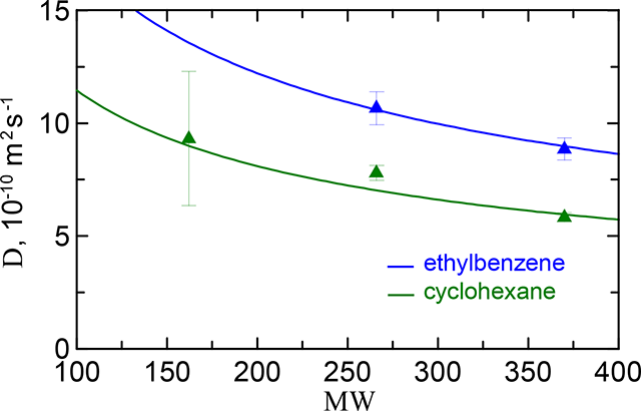
Diffusion coefficient of PS oligomers at infinite dilution in ethylbenzene and cyclohexane as a function of their molecular weight. The symbols mark the data from ref 16. The theoretical curve is eq 18 with Γ_ebz_ = 17.3 and Γ_chx_ = 11.5.

The theoretical values of *D*_*T*_ = *D*_*T*_(MW) for the PS solutions were determined similarly to the case of the alkane solutions considered above—according to eqs ([Disp-formula eq1]), ([Disp-formula eq2]), ([Disp-formula eq5])–([Disp-formula eq9]), and ([Disp-formula eq18]). The mass of the PS oligomers was determined within the interval MW_ebz_ ≤ MW ≤ 1000. The found values of *D*_*T*_ were fitted to eq ([Disp-formula eq17]). Three fitting parameters for PS are given in Table 3. In contrast to the alkanes, the value of the exponent turned out to be α = 1. Figure 8 shows the transition in the behavior of *D*_*T*_ from PS oligomers and short chains to the high polymer limit. The two dependencies for PS solutions in toluene and ethylbenzene almost coincide, which is a direct consequence of the proximity of the parameters of both solvents given in Table 2. Both dependencies are in excellent agreement with the experimental data of refs 11, 12, 15, 51–53 , over the *entire* range of polymer masses—from oligomers to the high polymer limit. We note in particular the nonobvious agreement in the case of monomers. In fact, if there is no thermodiffusion of the PS monomer in ethylbenzene, *D*_*T*_ = 0, then from formula ([Disp-formula eq17]) follows the relation Δ_*T*_MW_ebz_ = *a*η. This relationship is perfectly fulfilled if the values Δ_*T*_ and *a* from Table 3 are substituted for toluene and ethylbenzene. In the opposite case of long chains, the values of *ηD*_*T*_ approach the high polymer limit 6.6 · 10^–15^ N/K, which is very close to the universal plateau value η*D*_*T*_^*∞*^ ≈ 6 · 10^–15^ N/K observed in refs 15, 18 .

**Figure 8:**
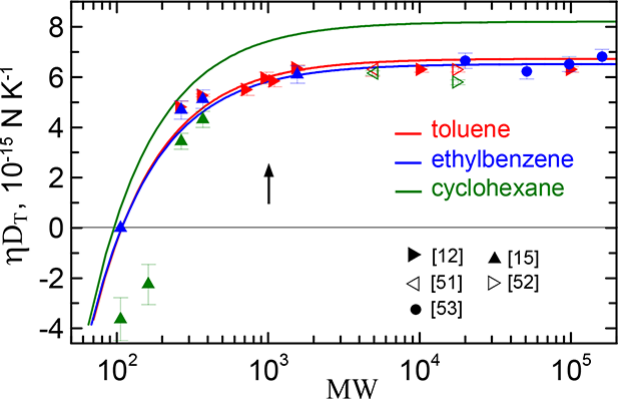
Product η*D*_*T*_ for PS in three solvents. The solid lines are the parametrization ([Disp-formula eq17]) with α = 1 and parameters from Table 3. The symbols are the experimental data. The inset shows the references of the data. The arrow marks the molecular weight of the Kuhn segment.

The predictions for PS in cyclohexane turn out to be the worst. This is mainly due to the wrong sign of *D*_*T*_ in the case of oligomers. We observed the similar behavior in ref 28 for cyclohexane-toluene mixture. According to ref 16, thermodiffusion of oligomers with weight 106 (ethylbenzene) and 162 takes place in the hotter layers of the mixture, *D*_*T*_ < 0. In contrast, the predictions indicate movement in the opposite direction—into colder regions. Accordingly, the whole curve *D*_*T*_ for PS in cyclohexane turns out to be shifted upward, leading to an overestimated limit η*D*_*T*_^*∞*^ ≈ 8.2 · 10^–15^ N/K.

How can the behavior of *D*_*T*_ in Figure 8 be understood qualitatively as the molecular weight of the polymer increases? To answer this question, we turn to the individual contributions to the Soret effect, similar to what was done above for alkanes. In Figure 9 these contributions are shown in the PS oligomer mass range up to the Kuhn segment mass in the case of PS in ethylbenzene, cf. with Figure 3. The following typical features of the behavior of the contributions can be drawn. (i) The dispersion and hard chain contributions compensate each other significantly, but in contrast to alkanes, the positive dispersion contribution dominates. The sum of both chemical contributions increases with molecular weight. (ii) The kinetic contribution also increases with MW. Thus, similar to alkanes, the chaining of PS segments leads to an increase of the Soret coefficient. (iii) For the smallest oligomer with mass equal to a monomer, thermodiffusion disappears in ethylbenzene. In Figure 9 this can be seen from the leftmost point, MW = MW_ebz_. From this we conclude that the entire *D*_*T*_ curve lies in the positive half of the plane: it starts at zero for the monomer case, increases with molecular weight at MW ≤ MW_Kuhn_, and saturates when the polymer becomes longer than the Kuhn segment. Since toluene is very close to ethylbenzene, this conclusion can also be applied to this solvent. As already noted, in the case of cyclohexane there is only a discrepancy in the initial value *D*_*T*_.

**Figure 9:**
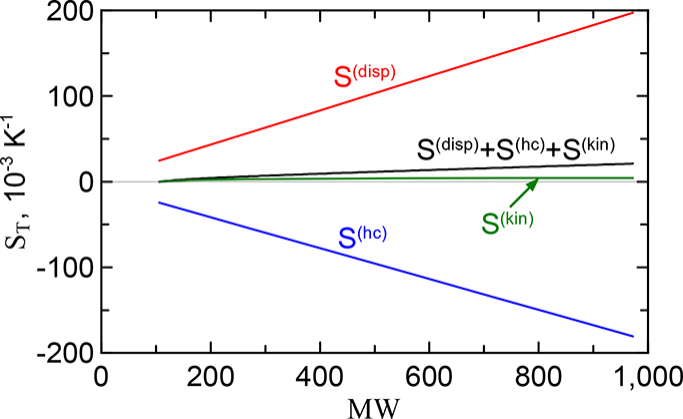
Full Soret coefficient and its individual contributions for PS oligomers with masses MW_ebz_ ≤ MW ≤ 1000 in ethylbenzene. The red, blue, and green lines mark the dispersion, hard-chain and kinetic contributions, respectively. The black solid line is the full Soret coefficient.

## Conclusions

In this paper we have theoretically studied the thermodiffusion of chain molecules as a function of their length, starting from oligomers and short chains up to high polymers. As polymers we consider polystyrene and polyethylene oligomers (alkanes), for which there is an extensive bibliography. Solvents are toluene, ethylbenzene, or cyclohexane. The polymers and solvents are chosen to be nonpolar and their solutions are assumed to be dilute.

The calculation of the Soret coefficient of the polymer is performed within the framework of the^28^ approach, where *S*_*T*_ is represented as the sum of the chemical and kinetic contributions. The chemical contribution is well described by the perturbed-chain statistical associating fluid theory, which characterizes the nonassociating and nonpolar pure fluids by three physically significant parameters: the segment number *m*, the hard-core segment diameter σ, and the segment–segment interaction parameter ϵ.^30^ For chained molecules, the parameters are determined according to the relations ([Disp-formula eq10])–([Disp-formula eq12]).^40,41^ For alkanes, the unknown coefficients are found by fitting to the PC-SAFT data.^33^ The coefficients for PS are obtained from boundary parameter values of the shortest (equal to one monomer) and infinite polymer chains. The calculated parameters are given in Table 1.

The kinetic contribution is described by eq 4, separating the gas term and the term associated with hydrodynamic fluctuations. The physically meaningful parameter of the kinetic contribution is the logarithmic temperature derivative of the solvent viscosity. It is known from rheological experiments. For three solvents this quantity is given in Table 2.

Figures 6 and 8 present the main results of our study. They show theoretical dependencies of the thermodiffusion coefficient *D*_*T*_ of dilute polymer solutions on the polymer molecular weight. Their remarkable common properties are the monotonic growth with molecular weight and the crossover from monomer to high polymer behavior. The growth is a result of segment chaining. The crossover is observed when the oligomers become of the order of the Kuhn segment.^14,15^ The Kuhn segment masses of flexible alkane molecules and rigid PS molecules differ by more than 4 times. Surprisingly, in the whole range of molecular weights the crossover for both polymers can be parametrized by the simple relation ([Disp-formula eq17]) with different values of the exponent α for alkanes and PS. The agreement between calculated and experimental values is particularly good for PS solutions in toluene and ethylbenzene. For alkanes in the same solvents, the predictions also describe the data well, but slightly overestimate them. More significant deviations between theoretical and measured results occur when cyclohexane is used as solvent. This behavior was actually expected, since it also occurs in the case of molecular mixtures with cyclohexane.

In this paper we have only dealt with infinitely diluted polymer solutions. An interesting objective will be to extend the treatment to finite concentrations. In order to tackle this task, it will be necessary to correctly address the friction mechanism that slows down thermodiffusion at finite concentrations. In refs 54,55 , it has been shown that the slowing down of *D*_*T*_ with increasing polymer concentration does not resemble the increasing macroscopic shear viscosity, which is mainly dominated by entanglements of longer chains and scales approximately as MW^3^. In ref 55, it is demonstrated that the concentration dependence of *D*_*T*_ is identical to that of the solvent self-diffusion coefficient. This leads to the conclusion that the friction relevant to *D*_*T*_ is the local friction acting on the length scale of a solvent molecule or polymer segment. The identical slowing down of both thermodiffusion and solvent self-diffusion at high polymer concentrations is a consequence of the approaching glass transition and is not related to chain entanglements at all.^54^ The latter are only relevant for the macroscopic shear viscosity as well as for the polymer self-diffusion coefficient. Semidilute solutions of long chains, e.g., already show a dramatic increase of the shear viscosity and a similar slowing down of polymer self-diffusion, whereas both solvent self-diffusion and thermodiffusion are still very close to their dilute solution limits.
